# Resin matrix ceramic crowns cemented on titanium bases: effect of surface treatments and stress distribution

**DOI:** 10.1590/0103-644020256140

**Published:** 2025-07-11

**Authors:** Mariana Itaborai Moreira Freitas, Mirelle Maria Ruggiero, Raissa Micaella Marcello-Machado, Vanessa Felipe Vargas-Moreno, Andréa Cândido dos Reis, Altair Antoninha Del Bel Cury

**Affiliations:** 1 Department of Prosthodontics and Periodontology, Piracicaba Dental School, University of Campinas(UNICAMP), Piracicaba, SP, Brazil; 2 School of Dentistry, Paulista University, Sao Paulo, SP, Brazil; 3 Department of Dental Materials and Prosthodontics, Ribeirão Preto Dental School, USP-University of São Paulo, Ribeirão Preto, Brazil

**Keywords:** aluminum oxide, CAD/CAM, titanium, ceramics

## Abstract

Using Titanium bases (TBs) has shown excellent results in implant-supported rehabilitation. However, failures due to decementation can occur, and bonding success depends on the TB surface treatment. This study evaluated the effect of different TB surface treatments on the retention and failure mode of resin matrix ceramic (RMC) crowns after thermocycling and the stress distribution of RMC cemented on the TBs. 120 TBs were divided into eight groups (n = 15) according to the material (resin nanoceramic (RNC) and polymer-infiltrated ceramic network (PICN)) and the surface treatment of the TBs: no treatment (NT); Airborne-particle abrasion with 50μm aluminum oxide (Al_2_O_3_) (AL); Airborne-particle abrasion with 30μm silica-modified Al_2_O_3_ particles (SIAL30) and Airborne-particle abrasion with 110μm silica-modified Al_2_O_3_ particles (SIAL110). After thermocycling, retention, and failure modes were analyzed. Retention data underwent two-way ANOVA and Tukey Post Hoc test (α=0.05). Finite element analysis (FEA) assessed stress distribution according to maximum principal stress (σ_max_) on the crown and maximum shear stress (τ_max_) on the cement layer. No significant difference was observed among the surface treatments or their interaction with the material (p > 0.05). However, RNC presented higher retention force values for all surface treatments (p<0.001). FEA showed a higher σ_max_ for PICN (317.31 MPa) than RNC (277.69 MPa), while τ_max_ was higher for RNC (246.54MPa) compared to PICN (213.55MPa). Most failures were cohesive in the material. In conclusion, surface treatments did not influence the retention of crowns to TBs, with the RNC having higher retention and better stress distribution for the crown.

## Resumo

A utilização de Bases de Titânio (BTs) tem apresentado excelentes resultados em reabilitações implanto-suportadas. Contudo, falhas por descimentação podem ocorrer e o sucesso da cimentação depende do tratamento de superfície das BTs. Assim, este estudo avaliou o efeito de diferentes tratamentos de superfície de BTs na retenção e modo de falha de coroas cerâmicas de matriz resinosa (CMRs) após termociclagem e a distribuição de tensões da CMRs cimentada sobre as BTs. Para isso, 120 BTs foram divididas em oito grupos (n = 15) de acordo com o material (resina nanocerâmica (RNC) e cerâmica infiltrada por polímero (CIP)) e o tratamento de superfície das BTs: sem tratamento (ST); Jateamento com partículas de óxido de alumínio de 50μm (Al_2_O_3_) (AL); Silicatização com partículas de Al_2_O_3_ modificadas com sílica de 30μm (SIAL30) e Silicatização com partículas de Al_2_O_3_ modificadas com sílica de 110 μm (SIAL110). Após a termociclagem, foram analisadas a retenção e modo de falha. Os dados de retenção foram submetidos à análise de variância de dois fatores (ANOVA) e teste post-hoc de Tukey (α=0,05). A análise de elementos finitos (AEF) avaliou a distribuição de tensão de acordo com a tensão principal máxima (σ_max_) na coroa e a tensão de cisalhamento máxima (τ_max_) na camada de cimento. Não foram observadas diferenças significativas entre os tratamentos de superfície ou sua interação com o material (p > 0,05). No entanto, a RNC apresentou valores de força de retenção mais altos para todos os tratamentos (p < 0,001). Na FEA, os valores de σ_max_ foram maiores para a CIP (317.31 MPa) em relação a RNC (277.69 MPa), enquanto os valores de τ_max_ foram maiores para a RNC (246.54 MPa) em comparação a CIP (213.55 MPa). A maioria das falhas foram coesivas no material. Em conclusão, os tratamentos de superfície não influenciaram na retenção das coroas às BTs, com a RNC tendo a maior retenção e melhor distribuição de tensões para a coroa.

## Introduction

Oral rehabilitation with implant-supported prostheses is considered a safe and reliable treatment for partially edentulous patients, with a survival rate of 95% after 5 years and 86.7% after 10 years of clinical follow-up ^1^. The gold standard treatment in single implant-supported rehabilitation is based on prefabricated screw-retained metal pillars, on which fabricated ceramic or metal-ceramic crowns restore the patient's function and aesthetics ^2^ ^,^ ^3^.

With the growth of the digital workflow, prefabricated titanium abutments, known as titanium bases (TBs), were developed to be used in implant-supported oral rehabilitations. Currently, these types of components have become popular and can also be used in analog flow ^4^. In this type of system, the crown is fixed to the abutment in a hybrid way ^2^, the TBs are screwed onto the implant, and the crown is cemented onto the TB. This cementation procedure can be performed in an extra-oral environment in the prosthetic laboratory or the dental office. This is advantageous because it facilitates and optimizes cementation work and prevents residual cement leakage around the implant platform, reducing the risk of biological complications, which may eventually induce marginal bone loss ^4^ ^,^ ^5^. Furthermore, compared to conventional abutments, these TBs have advantages, such as a highly accurate implant-abutment fit, manufacturing of excellent prosthetic parts, and a more faithful copy of the patient's emergency profile ^5^.

Monolithic crowns made by computer-aided design and computer-aided manufacturing (CAD/CAM) cemented onto titanium bases are a reliable option in the dental clinic, presenting excellent aesthetic properties and internal and marginal adjustments similar to crowns made by conventional impression ^6^. However, studies show that ceramics are friable materials and may be subject to premature failure, especially when subjected to fatigue in humid environments ^7^. In order to solve these limitations, resin matrix ceramics (RMCs) have been proposed as alternatives, and this category includes materials that have an organic matrix filled with ceramic particles ^8^.

RMCs are an alternative to conventional ceramics, offering a modulus of elasticity similar to dentin, along with greater ease of adjustment and repair compared to glass matrix ceramics ^8^ ^,^ ^9^. An example of RMC is resin nanoceramic (RNC), which contains dispersed nanoparticles of silica (~20 nm) and zirconia (4-11 nm), as well as nanoclusters of zirconium dioxide (0.6-10 μm) with 80 wt% ceramic nanoparticles. And polymer-infiltrated ceramic network (PICN), which is a hybrid ceramic consisting of a feldspathic ceramic matrix (86 wt%) infiltrated by an organic phase, containing UDMA and TEGDMA ^8^.

The RMCs have a higher polymer content; in addition, they are materials that tend to be less friable compared to glassy and polycrystalline ceramics ^8^ ^,^ ^9^. In a scenario of implant-supported rehabilitations, RMCs have a greater capacity to withstand loads, with a lower stress concentration in the peri-implant region. They are materials that tend to be less friable when compared to glass and polycrystalline ceramics ^10^. However, a systematic review that evaluated the clinical performance of crowns supported by all-ceramic implants ^3^ ^,^ ^5^, including those made from RMCs, showed that crown decementation failures could occur and the success of these restorations may be dependent on the interface bonding of titanium bases or abutments cemented on ceramic crowns ^5^.

To date, data in the literature refer to the conditioning protocol of cemented TBs on restorations made with zirconia and lithium disilicate ^2^ ^,^ ^5^. However, to our knowledge, no studies evaluated the best protocol for conditioning titanium bases cemented on crowns made with RMCs. In addition, another study analyzed the adhesion of titanium in flat disc-shaped specimens subjected to shear strength analysis ^8^, not considering the taper and mechanical interlocking of the different geometries of the titanium and abutment bases. Also, a recent study ^2^, presented results of pre-treatment of TBs evaluating the initial connection immediately after cementation and did not consider interface aging, which may interfere with adhesion and rehabilitation longevity.

Furthermore, there is no information in the literature on the biomechanical behavior of RMCs cemented onto TBs in the anterior region, considering a force applied in the direction of crown dislodgement. Since RMCs (RNC and PICN) are materials with different compositions ^8^, this factor may influence the stress distribution in the crown cemented onto the TBs, potentially affecting the longevity and success of the rehabilitation. This is crucial since the tensile stress can lead to flexural cracks, propagating upward and sideward, starting at the cementation interface surface of the crown ^11^.

Recognizing the need for scientific information on the cementation protocol for resin matrix ceramics cemented on titanium bases, as well as on the biomechanical behavior of the RNC and PICN on titanium bases, this study evaluated the different surface treatments of TBs on the retention and failure mode of resin matrix ceramic crowns after thermocycling and the stress distribution of RMC cemented on the TBs.

## Materials and methods

### Specimens preparation

One hundred and twenty TBs (2.0 x 4.0; SIN - Implant System, São Paulo, Brazil) were screwed into conical internal connection implants (Epikut; SIN - Implant System). The sets were embedded in acrylic resin (Jet; Dental Articles Classic Ltda, Campo Limpo Paulista, São Paulo, Brazil) surrounded by a PVC tube, using a dental surveyor (Delineador B2; Bio-Art, São Carlos, São Paulo, Brazil) to standardize the long axis alignment and parallelism between the acrylic base and the implants, following ISO 14801 for mechanical testing of dental implants ^12^. After polymerization of the acrylic resin, the TBs were tightened into the implants following the manufacturer's instructions (20 N·cm). The sample size was determined according to studies available in the literature ^5^ ^,^ ^13^.

Monolithic crowns of a maxillary central incisor were designed using CAD software, with a 2 mm hole in the center of the crown and a cement space of 70 μm between the crown and the TB. This design was developed to allow the pull-out analysis ^2^ ^,^ ^13^. These crowns were milled (Ceramill Motion 2; Amann Girrbach, Kobach, Austria) in two different materials (n = 60 / group): resin nanoceramic (RNC - Lava Ultimate; 3M Oral Care; St Paul, MN, USA) and polymer-infiltrated ceramic network (PICN - Enamic; Vita Zahnfabrik, Bad Sackingen, Germany).

The specimens were divided into eight groups (n = 15 / group) according to the RMC and the titanium-based surface treatment, as follows: No treatment (NT); Airborne-particle abrasion with 50μm aluminum oxide (Al_2_O_3_) (Aluminum Oxide; Bio-Art, São Carlos, SP, Brazil) (AL); Airborne-particle abrasion with 30μm silica-modified Al_2_O_3_ particles (CoJet Sand; 3M ESPE, St Paul, MN, USA) (SIAL30) and Airborne-particle abrasion with 110μm silica-modified Al_2_O_3_ particles (Rocatec Plus powder; 3M ESPE, St Paul, MN, USA) (SIAL110) ^5^.

The AL, SIAL30, and SIAL110 groups were sandblasted with particles at 2.8 bar for 20 seconds at a distance of 10 mm ^14^ until the metal turned a uniform dark color according to the manufacturer's recommendation. The device used for airborne particle abrasion in all groups was placed at a 45-degree angle ^15^. This is because the amount of silica (% by weight) deposited on the surface can be higher when the nozzle angle of the air abrasion device is held at 45 degrees to the surface compared to the 90-degree nozzle angle ^15^

After sandblasting, only the AL group was cleaned in an ultrasonic bath with distilled water for 10 minutes ^14^. After this step, the primer (Clearfil Ceramic Primer Plus; Kuraray Noritake Dental Inc, Okayama, Japan) was applied to all groups for 60 seconds and dried gently with a jet of air ^14^.

For the cementation of RMCs on titanium bases, ceramics were treated following the manufacturer's recommendations. RNC samples were sandblasted with aluminum oxide 50 μm grain size (Óxido de Alumínio; Bio-Art), at a distance of ∼1 cm for 5 s at 2 bar. After this step, the residues were removed in an ultrasonic bath with distilled water for 2 minutes and dried for 20 seconds, and the adhesive (ScotchbondTM Universal Adhesive; 3M Oral Care) was applied for 20 seconds and dried with an air jet for 5 seconds ^9^. On the other hand, the PICN was etched using 5% hydrofluoric acid (Condac porcelana; FGM Produtos Odontológicos Ltda, Joinville, SC, Brazil) for the 60s. After this step, the residues were removed in an ultrasonic bath with distilled water for 2 minutes and dried for 20 seconds followed by the primer (Clearfil Ceramic Primer Plus; Kuraray Noritake Dental Inc) application for 60 seconds, and gently dried with an air jet ^9^.

Then, crowns were cemented in the TB using dual resin cement (Panavia V5; Kuraray Noritake Dental Inc) ^13^. Excess resin cement was removed, and a 5 kg weight was applied for 10 minutes. Maintained under pressure, a glycerin gel was applied to the cementation line, and photopolymerization was carried out on the four faces of the crown with an LED light source (Valo Cordless; Ultradent, South Jordan, USA) for 20 seconds. After this step, the access holes for the screws and the hole in the bases were closed with polytetrafluoroethylene tape (Teflon; Chemours Co, Wilmington, Delaware), the primer was applied (Clearfil Ceramic Primer Plus; Kuraray Noritake Dental Inc) and in the final step, the conduit was filled with composite resin (Tetric EvoCeram A2; Ivoclar Vivadent, Schaan, Liechtenstein) and light curing was carried out in the cingulate region. The sets submerged in distilled water were stored in a moist environment at 37°C for 48 hours before testing. Afterward, the samples were thermocycling in water with a temperature range of 5°C to 55°C for 15,000 cycles with a dwell time of 30 seconds to artificially age the bonding interface (MSCT-3; Marcelo Nucci-ME, São Carlos, SP, Brazil) ^13^.

### Retention analysis and failure modes

**Figure 1:**
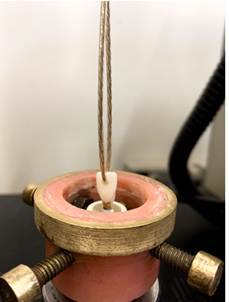
Demonstration of the retention test using a resin nanoceramic (RNC) crown, where a mixed failure occurred.

Retention forces were measured using a pull-out test in a universal testing machine (Model 4411; Instron, Canton, MA, USA) at a 0.5 mm/min crosshead speed. A metal wire 2 mm in diameter was threaded through the hole in the crown and attached to the testing machine's crosshead (Figure 1). The maximum retention force for each specimen was recorded in newtons (N) ^2^ ^,^ ^13^.

After the pull-out test, the surfaces of the TBs and the intaglio of the crowns were inspected under a polarized light microscope (AxioZoom V16; Zeiss, Oberkochen, Germany). Failure modes were recorded by a single observer and were classified into: Adhesive on TBs (cement remained predominantly on the crown (>90%)); Adhesive on the crown (cement remained predominantly on the TBs surface (>90%)); Mixed failure (cement remained on both TBs and crown surfaces) and Cohesive failure (Material failure). Representative specimens of each group were collected for fractographic analysis with scanning electron microscopy (SEM) (JSM-5600LV; Jeol, Boston, MA).

The average values obtained after the pull-out test were calculated using a statistical software program (IBM SPSS Statistics, v25; IBM Corp, Armonk, NY, USA). Normal data distribution was checked by descriptive analysis using skewness. Then two-way analysis of variance (ANOVA), and the Tukey post-hoc test was used to identify significant differences between groups, with a significance level of 5% (α=0.05).

Finite element analysis (FEA).

**Figure 2:**
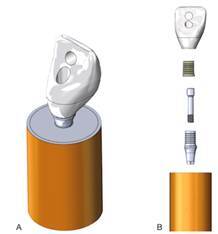
(A) Isometric view of the assembled model; (B) View of the crown, cement layer, TB, and TB screw.

The FEA was performed to evaluate the influence of the type of RMC on the stress distribution, using the same parameters applied in the *in vitro* test. Two groups were created only varying the crow material: RNC and PICN. For this, three-dimensional models of the cement layer (70 μm), acrylic base, and PVC tube were created in CAD software (SolidWorks 2024; SolidWorks Corporation, Concord, MA), being the same as described in the retention analysis. The CAD from the TBs, TBs screw, and implant were provided by the manufacturer. The crown CAD was the same as used in the retention analysis. The TBs were virtually screwed into the implant, and then the crowns were virtually cemented into the TBs (Figure 2). An assembly interference detection tool was then used.

After that, all the CAD models were imported into the FEA software (Ansys Workbench 15.0; Ansys, Inc, Canonsburg, PA, USA), where a convergence analysis (5% tolerance) with a tetrahedral mesh (0.6 mm elements size) was performed. The mechanical properties of each material component were introduced on the software (Table 1). All materials were considered homogenous, isotropic, and linearly elastic, and the contact conditions between all components were assumed as bonded ^16^.

**Table 1:** Materials mechanical properties.

Material	Elastic modulus (GPa)	Poisson’s ratio (()	References
RNC	12.7	0.45	Wendler et al. (17)
PICN	37.8	0.24	Wendler et al. (17)
Resin cement	18.3	0.33	Li-li et al. (22)
Titanium	104	0.34	Cruz et al. (23)
Acrylic resin	2.0	0.3	Darbar et al. (24)
PVC	1.43	0.4	Miniaci et al. (25)

The model was fixed in the base and a 289.17 N load (mean load of all groups studied) was applied at the crown hole to simulate the same conditions attended in the retention analysis. The crown was evaluated by maximum principal stress (σ_max_) and the cement layer by the maximum shear stress (τ_max_) ^9^. The results obtained for each criterion were quantitatively compared in megapascals (MPa). Also, a qualitative analysis was performed using the color pattern of the FEA images, which varied from warmer (red) to cooler (blue) tones, being the peak stress represented by the warmest tone.

## Results

**Figure 3:**
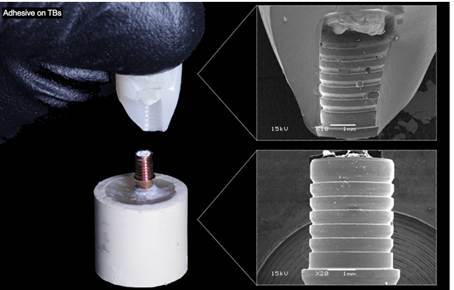
Adhesive failure on TBs - cement remained predominantly in the crown (>90%) after the pull-out test (RNC crown).

**Figure 4:**
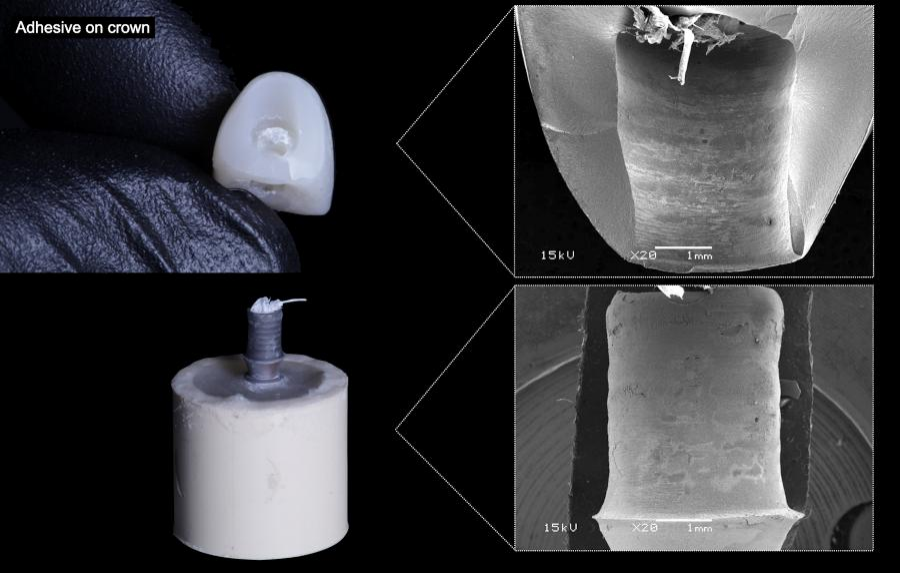
Adhesive failure on crown-cement remained predominantly on the TB surface (>90%) after the pull-out test (PICN crown).

**Figure 5:**
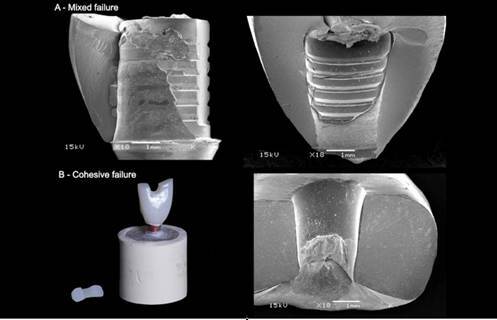
(A) Mixed failure - cement remained on the surface of the TBs and the crown surfaces after the pull-out test (RNC crown); (B) Cohesive Failure - Material Failure (PICN crown).

Two-way ANOVA indicated that no significant effect was attributed to surface and material-surface treatment interaction (p>0.05). However, the materials (RNC and PICN) showed a significant difference (p<0.05) (Table 2). Also, the recorded failure modes were represented in Table 3 according to each group, and the representative Scanning Electron Micrographs (SEM) of each type of failure (Adhesive on TBs, Adhesive on the crown, Mixed failure, and Cohesive failure) are represented in Figure 3, 4 and 5.

**Table 2:** Values of retention force (N) (mean standard deviation) for the different materials and surface treatments and Two-way ANOVA of the material, surface treatment, and interaction between these variables.

Surface Treatment	Material		ANOVA	F-value	*P*
	RNC	PICN			
NT	298.61±47.01	270.48±28.61	Material	40.297	<0.001*
AL	328.04±44.84	274.45±35.06	Surface Treatment	2.097	0.106
SIAL30	331.46±44.89	261.12±24.48	Interaction	1.210	0.310
SIAL110	301.80±57.37	247.40±43.90			

*Statistically significant difference (p < 0.05).

**Table 3:** Type of Failure Modes after pull-out testing.

Surface Treatment	Material	Failure Mode			
		Adhesive on TBs	Adhesive on crown	Mixed	Cohesive
NT	RNC	5	0	1	9
	PICN	0	0	0	15
AL	RNC	1	6	0	8
	PICN	0	8	0	7
SIAL30	RNC	0	5	2	8
	PICN	0	4	0	11
SIAL110	RNC	0	7	2	6
	PICN	0	4	1	10

**Figure 6:**
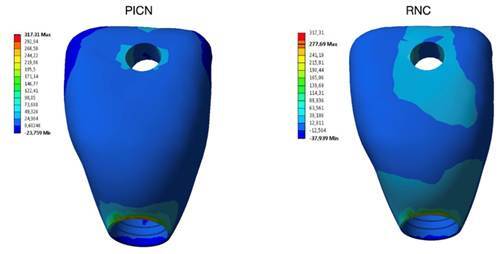
Peak stress concentration in crowns made of PICN and RCN.

**Figure 7:**
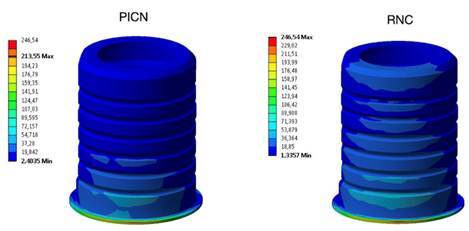
Peak stress concentration in the cement layer in the groups of crowns made of PICN and RCN.

The FEA assessment results are described in Table 4. The RNC group exhibited better stress distribution for the crown (277.69 MPa) but presented a higher stress value for the cement layer (246.54). Regarding the peak stress concentration area, all peak stress was the same in both groups. The crown σ_max_ (Figure 6) and the cement layer τ_max_ peak stress (Figure 7) were located in the cementation area of ​​the interface on the buccal side of the crown.

**Table 4:** Maximum principal stress (σ_max_) (MPa) for the crown and maximum shear stress (τ_max_) (MPa) for the cement layer.

	RNC	PICN
Crown (σ_max_)	277.69	317.31
Cement layer (τ_max_)	246,54	213,55

## Discussion

Understanding how to perform a better cementation protocol for resin matrix ceramics crowns cemented on titanium bases is essential to prevent clinical failures ^5^. This study evaluated the effect of different surface treatments of TBs cemented onto RMC crowns on retention and failure mode after thermocycling. The different surface treatments would not influence the retention and failure mode of resin matrix ceramic crowns; However, although the different surface treatments on the TBs and the interaction between material and treatment did not present statistical differences, there was a difference between the two materials.

In the present study, RNC presented higher retention force values for all surface treatments performed compared to PICN, with a statistically significant difference between the materials. Most of the failures observed in all groups were cohesive, and this is related to material strength. PICN is a hybrid ceramic comprising a sintered glass-ceramic network including 58-63% SiO_2_, 20-23% Al_2_O_3_, 9-11% Na_2_O, 4-6% K_2_O and 0.1% ZrO_2_ (86% by weight) containing a methacrylate polymeric network with UDMA and TEGDMA (14% by weight). In comparison, RNC is a highly cured resin matrix reinforced with silica (20nm) and zirconia (4-11nm) nanoparticles, and ZrO_2_ nanoclusters with 80% by weight of ceramic nanoparticles, and its organic part contains Bis-GMA, UDMA, Bis-EMA, TEGDMA ^8^ ^,^ ^9^. As RNC has a lower modulus of elasticity (12.7 GPa) ^17^ when compared to PICN (37.8 GPa) ^17^, this material better dissipates stress, withstanding more stress before failure, which may result in higher retention values compared to PICN ^18^. These findings corroborate with the study by Rizzatto et al. (2023) ^19^ and Wendler et al (2020) ^20^, in which although flexural strength was used, RNC showed better results than PICN. This result was attributed to the fact that RNC has a higher polymer content, and better absorbing applied loads, thus increasing the material's resistance to fracture.

Regarding the surface treatment, all three methods used in the study (AL, SIAL30, and SIAL 110) did not show statistical significance between them. Studies have shown ^21^, that after airborne-particle abrasion, surface roughness increases, which is important to increase microretentions on the surface of TBs and improve the cementation condition of the prosthetic crown to the TB. Furthermore, the silica deposited on the surface of the TBs in SIAL groups also promotes a chemical adhesion after the application of the primer (Clearfil Ceramic Primer Plus; Kuraray Noritake Dental Inc). The primer used contains 10-methacryloyloxidecyl dihydrogen phosphate (10-MDP), this monomer has a phosphate ester group, which bonds directly to the metal oxides present in the TBs and a methacrylate group, which bonds to the resin cement ^5^. Furthermore, the primer also has a silane coating agent in its composition, which bonds to the resin cement and the silica contained in the metal oxides after blasting with SiO_2_-Al_2_O_3_ particles, increasing bond strength after cementation ^21^.

In the SIAL groups, the ultrasonic bath was not used because a recent study ^21^, demonstrated that the silica deposited after airborne-particle abrasion with SiO_2_-Al_2_O_3_ particles could be removed after cleaning methods, compromising the bond strength. Even though the surface was not cleaned after sandblasting, none of the study groups showed a statistically significant difference in retention force after the pull-out test. This could be because thermocycling reduced the chemical bond provided by silica-silane and resin cement-10-MPD after cementation. Additionally, the titanium bases have pre-fabricated retentions in their macrogeometry, which may have aided in mechanical retention, resulting in no statistically significant difference between the sandblasting methods employed in the study.

In relation to the failure mode, the adhesive failure on the crown was the second most reported failure in which the cement remained predominantly on the TB surface (>90%). This result shows that the chemical and mechanical bond between the TBs and the cement was stronger in groups treated by airborne-particle abrasion (SIAL110, SIAL30, and AL) than the other groups due to the surface treatments performed, which may have increased the surface roughness. However, in this failure mode, the bond between the crown and the cement was weak, considering the failure mode results after the retention test. It can be because RMCs are polymerized at high pressure and temperature, leading this material to have a low number of unreacted monomers available to copolymerize with composite cement, damaging the adhesion between the cement and the material ^18^. Extrapolating the study's results, we can raise the hypothesis that by improving surface treatment and adhesion between RMCs, we would have more cohesive failures, but this will depend on the resistance of the material used in the rehabilitation.

Regarding the FEA, the analysis was conducted using the same parameters as those applied in the *in vitro* tests. This approach allowed for a direct comparison between the results of the retention and failure mode analysis and the areas of greatest stress concentration identified in the prosthetic structure, thereby enabling validation of the findings. The results obtained in the FEA corroborate the findings of the retention test. Concerning the crown, PICN exhibited a higher σ_max_ value (317.31 MPa) compared to RNC (277.69 MPa). This can be explained because PICN has a higher modulus of elasticity, consequently, this material accumulates more stress within its structure compared to the RNC crown. These results are consistent with the failure mode, as PICN exhibited more cohesive failures (71.66%) compared to RNC (51.66%). As for τ_max_ evaluated in the cement layer, RNC showed a higher stress concentration at the restoration cement interface (513.28 MPa) compared to PICN (482.58 MPa). This phenomenon can be attributed to the lower modulus of elasticity of RNC compared to PICN, resulting in reduced stress concentration in the crown but increased stress accumulation in the cement layer, which may elevate the likelihood of adhesive failures relative to PICN. Regarding failure modes, although cohesive failure was predominant in RNC, this material demonstrated a higher incidence of adhesive and mixed failures (48.33%) compared to PICN (28.33%). These observations align with the findings of the FEA analysis and provide mutual validation.

In this study, the samples were only subjected to the thermocycling aging test, with chewing force not being evaluated as in a clinical scenario. One limitation of the study was that only one height of TBs was tested, and as there are different heights available on the market, this factor may influence retention and the results obtained. Also, it is important to highlight that the FEA was used in this study as a reliable approach to evaluate the biomechanical behavior of the crown material, being able to assess the location of peak stress and correlate it with the *in vitro* results. Also, it is recommended that future investigations consider testing different surface treatments on RMCs to improve adhesion between this material and the TBs. Furthermore, prospective clinical investigations are necessary to investigate and confirm these current in vitro findings.

Within the limitations of this study, it is concluded that the different TB surface treatments did not influence the retention between the TBs and crowns, with the RNC presenting higher retention and better stress distribution for the crown.
